# Aflatoxin Contamination in the Maize Supply Chain: A Case of Nampula Province, Mozambique

**DOI:** 10.3390/toxins18100421

**Published:** 2026-09-30

**Authors:** Ibraimo Teleha Chabite, Jamal Bakari Kussaga, Frida Nyamete, Quenton Kritzinger, Shivan Maharaj, Rashid Suleiman

**Affiliations:** 1 Laboratory of Quality and Food Safety (LQSA), Lúrio Interdisciplinary Study Center (CEIL), Lúrio University, Nampula P.O. Box 364, Mozambique; 2 Department of Food Science and Agro-Processing, Sokoine University of Agriculture, Morogoro P.O. Box 3006, Tanzania; kussaga@sua.ac.tz (J.B.K.); frida.nyamete@sua.ac.tz (F.N.); rashid@sua.ac.tz (R.S.); 3 Department of Plant and Soil Sciences, University of Pretoria, Pretoria 0028, South Africa; quenton.kritzinger@up.ac.za; 4 Mass Spectrometry, Central Analytical Facilities, University of Stellenbosch, Private Bag X1, Matieland 7600, South Africa; shivanm@sun.ac.za

**Keywords:** aflatoxin, maize, value chain, food safety, Mozambique

## Abstract

Aflatoxin contamination in maize remains a major food safety and public health concern in Nampula Province, Mozambique. Maize samples obtained from farmers, traders, and milling units across the districts of Malema, Mossuril, Mecuburi, Monapo, and Nampula in 2024 and 2025 were analysed using UPLC-ESI-MS/MS. In 2024, aflatoxins were detected in samples from Mecuburi (43%), Mossuril (17%), Monapo (17%), and traders (13%), with some exceeding the regulatory limit of 15 µg/kg. AFB_1_ was the predominant aflatoxin, followed by AFB_2_. At the farmer level, Mecuburi recorded the highest contamination, with AFB_1_ and AFB_2_ detected in 43% of samples and AFG_1_ and AFG_2_ in 29%, while Mossuril had 20% contamination with AFB_1_ and AFG_1_. The maximum concentration was 4221.33 µg/kg of AFB_1_ in Mecuburi, whereas the minimum detected concentration was 2.82 µg/kg of AFB_2_. Aflatoxins were not detected above the limit of detection in milling samples or in samples collected in 2025. Despite the relatively low prevalence, the occurrence of extremely high aflatoxin concentrations highlights persistent contamination risks and the need for integrated mitigation strategies throughout the maize supply chain.

## 1. Introduction

Aflatoxins (AFs) are secondary metabolites produced by the genus *Aspergillus* [1]. *Aspergillus* section *Flavi* has a significant impact on human health, agriculture, the food industry, and trade [2]. Climatic conditions and agronomic practices in sub-Saharan Africa greatly favour the development of *Aspergillus* section *Flavi* and the consequent production of AFs in food [3]. AFs are largely produced by *Aspergillus flavus* and *Aspergillus parasiticus* [1]. More than 18 types of AFs are known, and the most toxic for public health have been aflatoxins B_1_, B_2_, G_1_, and G_2_ (AFB_1_, AFB_2_, AFG_1_, and AFG_2_) [4]. AF exposure has been linked to liver cancer, immunosuppression, stunting in children due to chronic effects, and sudden deaths due to acute effects [5,6]. However, the effects depend on the duration of exposure, exposure to other toxins, dose of ingestion, age, gender, health and nutritional status, and animal species [7]. AFB_1_ is among the most potent carcinogens for public health and is classified as a group 1 human carcinogen [8]. Value chains, particularly cereals, legumes and oilseeds, may experience market restrictions due to AF contamination [9]. Several countries have established regulatory limits for AFB_1_ and total aflatoxins. The Codex Alimentarius Commission has set maximum permissible levels of 15 µg/kg for maize and 10 µg/kg for maize-derived products [10].

Maize is a staple food in several African countries, including Mozambique [11,12]. Maize farming occupies 1/3 of the total cultivated area in Mozambique, producing about 2.0 million metric tons [13]. Smallholder farmers contribute to more than 80% of total production [14]. Although maize grows in all agro-ecological zones (AEZ) of Mozambique, northern and central regions are the largest maize producers, with a combined national output of approximately 3 million tons between 2014 and 2018 [14].

Typical of agricultural value chains in Africa, the maize chain involves various actors. The chain is dominated by informal traders and small processors who may lack requisite knowledge for proper handling [15]. AF contamination remains widespread in Mozambique’s food chain, posing serious risks to health, food security, and trade [4]. AF contamination in maize is a serious and ongoing food safety concern in Nampula Province, which has been identified as an “aflatoxin hotspot” due to favourable climatic conditions such as high temperatures, drought stress, and poor post-harvest handling that promote fungal growth [4]. Poor drying practices, delayed harvesting, and inadequate storage conditions, such as high moisture and use of traditional storage structures, further increase aflatoxin accumulation [16]. Many smallholder farmers in Nampula lack access to proper drying technologies and awareness of aflatoxin risks, allowing contaminated grain to enter the market. As maize moves to traders, the risk becomes amplified through the aggregation and mixing of grains from different sources. Traders often prioritise volume and price over quality without systematic testing. Contaminated maize is mixed with clean batches, spreading aflatoxins throughout the supply chain. Weak regulatory enforcement and limited monitoring systems in Mozambique make it difficult to detect and remove contaminated lots at this stage [17]. During milling, standard processing methods do not destroy AFs, so contaminated maize is converted into flour and other products that still contain toxins [18]. Studies in Mozambique have detected aflatoxins in maize flour at levels that pose a potential long-term health risk, particularly because maize is a staple food consumed daily [19]. This creates a continuous exposure pathway for the population. For consumers, especially in rural and low-income households in Nampula, the danger is significant because maize is consumed frequently and often stored at home under suboptimal conditions, allowing further fungal growth.

Hence, the focus of this study is to track aflatoxins throughout the maize supply chain, showing the level of contamination among farmers, traders, and mills from different districts of Nampula Province, Mozambique, between two consecutive years (2024 and 2025), using UPLC-ESI-MS/MS to address gaps in the limited research available in this field.

## 2. Results

### 2.1. Farmers’ Aflatoxin Results

The study showed that in 2024, 21% of the farmers’ samples were contaminated with AFs. Mecuburi exhibited the highest prevalence of aflatoxin contamination among farmers, with AFB_1_ and AFB_2_ detected in 43% of samples and AFG_1_ and AFG_2_ in 29%. Furthermore, 43% of samples exceeded aflatoxin concentrations of 5 µg/kg and 15 µg/kg, respectively (Table 1). Monapo and Mossuril had 17% of their samples with aflatoxin levels above 15 µg/kg (Table 1).

**Figure 1:**
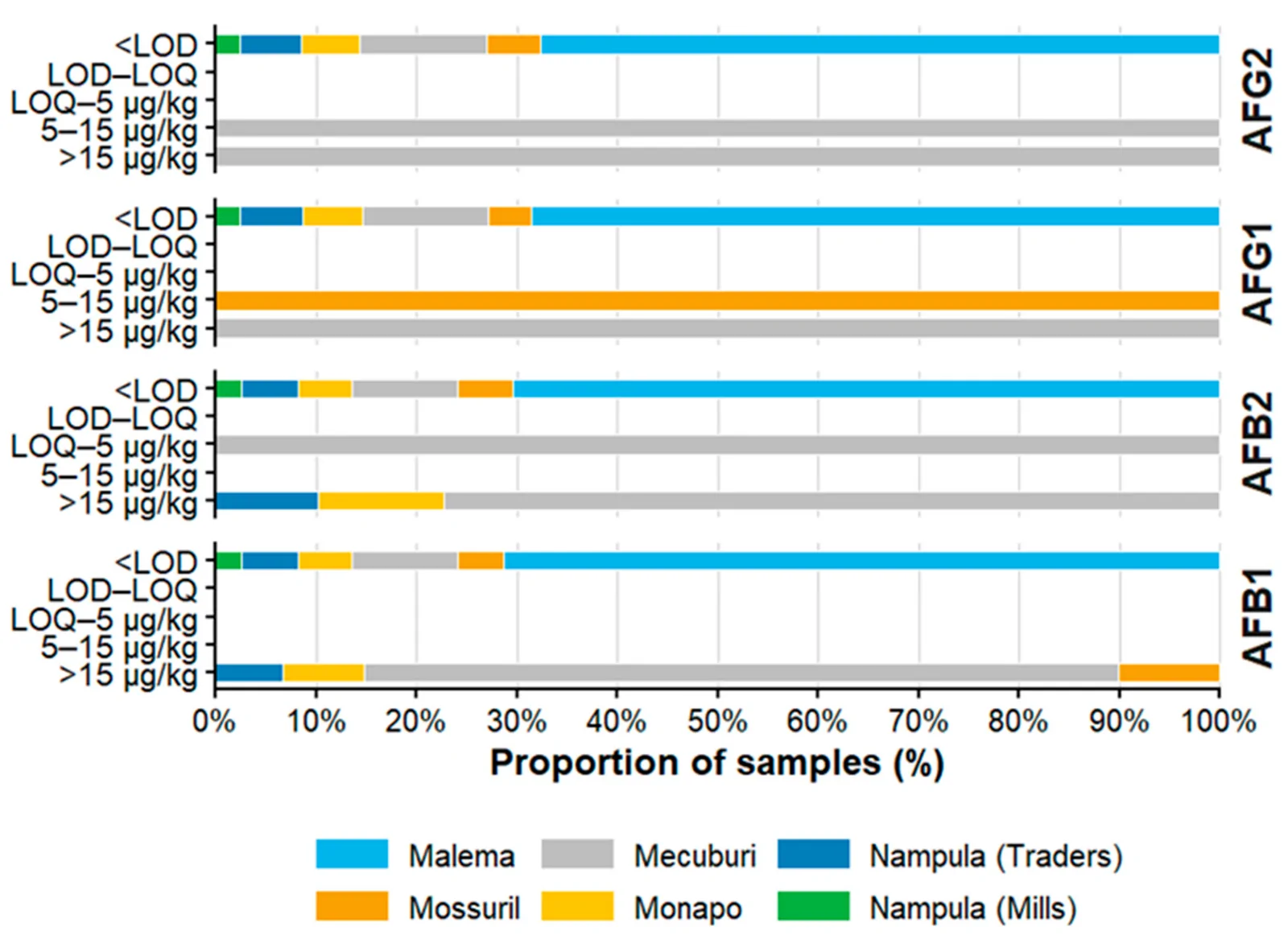
Farmers’, traders’, and mills’ samples distribution (%) according to the level of AF contamination. LOD = limit of detection; LOQ = limit of quantification.

The distribution of AFB_1_, AFB_2_, AFG_1_, and AFG_2_ contamination levels among the districts revealed that the majority of samples were below the LOD, with Malema recording 100% of samples below the LOD for all aflatoxins analysed (Figure 1).

In 2024, all aflatoxins were below the LOD in maize from Malema farmers. The highest AF contamination level detected was for AFB_1_, reaching 4221.33 µg/kg in Mecuburi. The lowest AF level detected was 2.82 µg/kg for AFB_2_ in Mecuburi (Table 2). Mecuburi is the district where the four types of AF (B_1_, B_2_, G_1_, and G_2_) were detected. The predominance of values below the LOD explains the low median values (0.50 µg/kg) despite the occurrence of extremely high concentrations in some positive samples, particularly for AFB1 in Mecuburi and Monapo (Table 2).

**Table 2:** Aflatoxin concentration in farmers’, traders’, and mill maize samples from two different years.

Year	Stakeholder	District		Aflatoxin Concentrations (μg/kg)			
				B_1_	B_2_	G_1_	G_2_
2024	Farmers	Malema	Positive samples	0	0	0	0
			Median (IQR)	0.5 (0.0)	0.5 (0.0)	0.5 (0.0)	0.5 (0.0)
			Range	<LOD	<LOD	<LOD	<LOD
		Mossuril	Positive samples	1	0	1	0
			Median (IQR)	0.5 (0.0)	0.5 (0.0)	0.5 (0.0)	0.5 (0.0)
			Range	19.91	<LOD	8.96	<LOD
		Mecuburi	Positive samples	3	3	2	2
			Median (IQR)	0.50 (61.74)	0.50 (2.32)	0.50 (91.27)	0.50 (75.00)
			Range	38.43–4221.33	2.82–187.66	183.29–730.80	8.95–150.00
		Monapo	Positive samples	1	1	0	0
			Median (IQR)	0.50 (0.00)	0.50 (0.00)	<LOD	<LOD
			Range	1940.66	133.38	<LOD	<LOD
	Traders	Nampula	Positive samples	1	1	0	0
			Median (IQR)	0.50 (0.00)	0.50 (0.00)	<LOD	<LOD
			Range	201.70	38.75	<LOD	<LOD
	Mills	Nampula	Positive samples	0	0	0	0
			Median (IQR)	0.5 (0.0)	0.5 (0.0)	0.5 (0.0)	0.5 (0.0)
			Range	<LOD	<LOD	<LOD	<LOD
2025	Farmers	Malema	Positive samples	0	0	0	0
			Median (IQR)	0.5 (0.0)	0.5 (0.0)	0.5 (0.0)	0.5 (0.0)
			Range	<LOD	<LOD	<LOD	<LOD
		Mossuril	Positive samples	0	0	0	0
			Median (IQR)	0.5 (0.0)	0.5 (0.0)	0.5 (0.0)	0.5 (0.0)
			Range	<LOD	<LOD	<LOD	<LOD
		Mecuburi	Positive samples	0	0	0	0
			Median (IQR)	0.5 (0.0)	0.5 (0.0)	0.5 (0.0)	0.5 (0.0)
			Range	<LOD	<LOD	<LOD	<LOD
		Monapo	Positive samples	0	0	0	0
			Median (IQR)	0.5 (0.0)	0.5 (0.0)	0.5 (0.0)	0.5 (0.0)
			Range	<LOD	<LOD	<LOD	<LOD
	Traders	Nampula	Positive samples	0	0	0	0
			Median (IQR)	0.5 (0.0)	0.5 (0.0)	0.5 (0.0)	0.5 (0.0)
			Range	<LOD	<LOD	<LOD	<LOD
	Mills	Nampula	Positive samples	0	0	0	0
			Median (IQR)	0.5 (0.0)	0.5 (0.0)	0.5 (0.0)	0.5 (0.0)
			Range	<LOD	<LOD	<LOD	<LOD

Note: LOD = limit of detection (as set in Table 3). Values below the LOD were assigned 0.5 µg/kg (LOD/2) for statistical analysis. The median should not be interpreted as indicating that contaminated samples had concentrations of 0.5 µg/kg; rather, it reflects the predominance of non-detects in the group. IQR = interquartile range. The detected range includes only samples with concentrations ≥ LOD.

**Table 3:** Validation parameters obtained for the method.

Aflatoxin	LOD (µg/kg)	LOQ (µg/kg)	Linearity (R^2^)	% Recovery (80–120)			Intra-Day (Inter-Day *) Precision (RSD; ≤20%)		
				7 (µg/kg)	70 (µg/kg)	700 (µg/kg)	7 (µg/kg)	70 (µg/kg)	700 (µg/kg)
B_1_	0.3	1.0	0.999	100.14	95.21	88.78	3.50 (3.23)	2.05 (2.02)	1.84 (4.49)
B_2_	0.3	1.0	0.999	107.57	106.09	89.28	3.64 (4.26)	1.77 (2.34)	3.15 (4.52)
G_1_	0.3	1.0	0.999	106.86	100.41	88.24	4.86 (4.60)	1.83 (1.52)	1.40 (3.94)
G_2_	0.5	2.0	0.999	108.29	99.06	89.93	9.36 (11.32)	1.19 (2.20)	1.59 (3.83)

* Note: Inter-day precision for the QC samples is shown in brackets.

The results show that there was no statistically significant difference (*p* > 0.05) between the four Nampula districts (Malema, Mossuril, Mecuburi, and Monapo) and AF concentrations (B_1_, B_2_, G_1_, and G_2_) in farmers’ maize samples from the 2024. All 2025 farmers’ samples were below the LOD.

### 2.2. Farmers’ Moisture Results

In 2024, the initial moisture content for maize samples ranged from 17.80% to 12.80%, with a mean of 17.02 ± 0.74% to 13.94 ± 1.42%. Final moisture content for maize samples ranged from 14.00% to 10.80%, with a mean of 12.63 ± 1.08% to 12.06 ± 0.80% (Table 4). Monapo showed the highest mean initial moisture content (17.02%), followed by Mecuburi (16.67%) (Table 4).

In 2025, the mean initial moisture content varied from 16.98 ± 0.79% in Mossuril to 14.19 ± 1.08% in Mecuburi, while observed values ranged from 10.30% in Malema to 17.70% in Monapo. For final moisture content, the mean values ranged from 12.98 ± 1.34% in Malema to 12.00 ± 0.78% in Mossuril, with individual values ranging from 10.30% to 15.40%. Mossuril and Monapo recorded the highest mean initial moisture contents, at 16.98 ± 0.79% and 16.70 ± 1.10%, respectively (Table 4). These differences may be associated with the geographical location of the districts within Nampula Province.

Mossuril is in the coastal zone, whereas Mecuburi and Malema are inland, with Monapo occupying an intermediate position. These contrasting geographical settings are associated with variations in relative humidity, which may influence the moisture content of harvested maize.

Maize moisture content intended for storage should be between 11% and 13%. Final moisture content remained close to 12–13% in most districts in both years, despite variation among individual samples (Table 4). Malema had the highest mean final moisture in 2025 (12.98 ± 1.34%), while Mossuril had the lowest (12.00 ± 0.78%). Compared with the initial moisture content, final moisture was consistently lower across all districts, indicating substantial moisture reduction during the post-harvest drying process.

### 2.3. Farmers’ Aflasafe Use

In all districts where the samples were collected, only Malema had 20% of fields treated with Aflasafe. Among these fields, 10% were treated in the last 3 years and 10% in the last year.

### 2.4. Traders’ and Mills’ Results

For traders and mills in 2024, aflatoxin contamination was observed exclusively in maize samples collected from traders, with 13% testing positive for both AFB_1_ and AFB_2_. Furthermore, 13% of the traders’ samples contained aflatoxin levels exceeding the regulatory limits of 5 µg/kg and 15 µg/kg (Figure 1). The highest and lowest levels detected were 201.70 µg/kg for AFB_1_ and 38.75 µg/kg for AFB_2_, respectively (Table 2). The average for AFB_1_ was 22.86 µg/kg, and the average for AFB_2_ was 4.75 µg/kg. No AF was detected in traders’ samples from 2025; all were below LOD (Figure 1). However, no AF was detected in mill samples in either year (2024 and 2025); all samples were below the LOD (Figure 1).

## 3. Discussion

AF contamination in food across the value chain has been a challenge for stakeholders, particularly in Sub-Saharan Africa. AF contamination in maize can occur at any stage: pre-harvest, harvesting, and post-harvest (transportation and storage) [20,21]. At the farmer level, the risk begins in the field where maize can become infected before harvest, especially under drought or pest stress [12]. The study found that most maize samples collected from farmers, traders, and millers in 2024 had aflatoxin concentrations below the LOD. Although the proportion of contaminated samples was relatively low, the concentrations of AFB_1_, AFB_2_, AFG_1_, and AFG_2_ detected in samples from farmers and traders were considerably high. Climatic conditions, particularly high temperatures and elevated humidity, are well-recognised factors that promote the growth of aflatoxigenic fungi and the subsequent accumulation of AFs in maize [22]. Although Malema and Mecuburi belong to the same AEZ R7, they have different climate conditions. Mecuburi has a sub-humid climate with annual rainfall between 800 and 1200 mm, and is closer to R8. Monapo and Mossuril are part of R8, and the average temperature exceeds 25 °C. The climate characterisation in the R8 and some R7 zones may facilitate the growth of AF-producing fungi in crops due to hotter conditions and drier periods [18]. Some of the farmers’ fields (20%) in Malema applied biocontrol (Aflasafe) and were located in the R10 zone, which may also influence the results, although the study is not conclusive and further data are still required. Aflasafe is highly effective in reducing aflatoxin accumulation in maize [18,23]. Generally, in Mozambique, AF levels are low in the highlands (>600 mm altitude), characteristic of the R10 zone [24].

The annual climate summary for Nampula Province shows a slight difference between 2024 and 2025 that may affect AF accumulation. In 2024, the temperature maximum average was 30–35 °C in the hot season, rain concentrated between January and March, and drier mid-year conditions, while in 2025, the temperature maximum average was 30–34 °C, a seasonal rainfall pattern, and a typical tropical savannah climate [25,26]. The interannual difference should also be considered in relation to the broader climatic conditions affecting Nampula Province. The 2023/24 agricultural season was influenced by El Niño, with irregular rainfall, dry periods and elevated temperatures reported in parts of Mozambique, including rainfall deficits in Nampula [27]. The early rains from October and November were followed by long dry spells and above-average temperatures, resulting in the loss of most of the early planted crops and forcing households to replant [28]. In contrast, the subsequent 2024/25 season experienced a different rainfall regime, including substantial rainfall events and tropical-cyclone activity in northern Mozambique [27]. The annual rainfall averaged 727 mm in 2024 and 1355 mm in 2025 [25]. These differences may have altered plant water stress, kernel susceptibility to *Aspergillus* infection and post-harvest drying conditions. Nevertheless, climate should not be considered the sole explanation for the observed reduction in aflatoxin contamination. Relative frequency of *Aspergillus flavus* morphotypes and diversity vary among years, seasons, regions, cultivation areas, and crops [29,30]. In addition to weather conditions, prolonged storage of maize samples from 2024 before analysis may have influenced the AF levels. AFB_1_ levels in Mecuburi, Mossuril, Monapo, and among traders exceeded 15 µg/kg. The highest levels of AFB_1_ detected were 4221.33 µg/kg among farmers and 201.70 µg/kg among traders. AFB_1_ has been linked to liver cancer, stunting in children, and other diseases in Mozambique [31,32] in cases of chronic exposure. Because one in two children is stunted, the future productive and intellectual capacity of half of Mozambicans may decrease, affecting human development [33]. The International Agency for Research on Cancer (IARC) categorised AFB_1_ as a carcinogenic toxin of group 1 [8]. As a member of Codex Alimentarius, Mozambique adopted 10 µg/kg as the maximum total aflatoxin limit for peanuts, cassava, rice, and beans, 15 µg/kg for maize, and AFB_1_ not exceeding 5 µg/kg in food [4,34]. To date, no cases of acute aflatoxicosis associated with consuming AF-contaminated food have been documented in Mozambique. However, the AFB_1_ and AFG_1_ detected in these studies indicate a potential risk of severe health effects if the contaminated maize were consumed. Maize flour blended with groundnut is commonly used as a nutrient-rich food for children. However, both maize and groundnut are highly susceptible to fungal infestation and subsequent AF contamination [18]. AFB_1_ and AFG_1_ are more toxigenic than AFB_2_ and AFG_2_. A study conducted in Gaza Province, southern Mozambique, found total AF levels in maize to be 9200.0 µg/kg in Chokwe, 1950.0 µg/kg in Manjacaze, and 8736.0 µg/kg in Chongoene [34]. High AF levels are not confined to Mozambique; they are also found in other African countries, Kenya and Tanzania, which have a record of outbreaks [35,36]. A study in Togo found an AF level of 1600 µg/kg in a maize sample from the Region des Plateaux [37]. In Zimbabwe, the maximum AF level found in maize was 1369 µg/kg [12]. Many African countries invest millions of dollars in health care costs due to AF-related cancers [35].

Besides posing serious public health issues, AF contamination of maize and maize derivatives also presents a significant economic barrier in low- and middle-income countries [38], reducing income for all stakeholders in the supply chain [23]. High levels of AFs prevent traders from exporting or selling maize at fair market price, forcing them to sell on the domestic market, where enforcement of legislation is weak. These occur in large African countries where AF regulations are not enforced [39]. The rejection of groundnuts in the European Union market causes economic losses for farmers in Mozambique [18]. In many instances, maize faces a higher risk of aflatoxin contamination at the trader level due to a combination of factors associated with transportation and storage practices. Substantial economic losses have been reported in several African countries, largely attributable to trade restrictions and market rejection of agricultural commodities exceeding permissible AF limits [40].

A substantial proportion of the maize samples collected in 2024, and all samples collected in 2025, had aflatoxin concentrations below the LOD, indicating that no aflatoxins were detected. Similar findings have been reported in Mozambique, where maize samples also contained aflatoxin levels below the LOD of 1.0 μg/kg [34]. Likewise, a study conducted in Nampula Province found that aflatoxin concentrations in maize flour obtained from mills were consistently below 1.0 μg/kg, with concentrations ranging from 0.25–0.33 μg/kg for AFB_1_, 0.02–0.24 μg/kg for AFB_2_, 0.27–0.36 μg/kg for AFG_1_, and 0.01–0.12 μg/kg for AFG_2_ [19]. Comparable results have also been reported in Tanzania, where only a small proportion of maize samples contained detectable levels of aflatoxins [36]. Mills’ information access, which facilitates good management practices, such as buying high-quality maize, cleaning and sorting, proper transport, and good storage facilities, may have contributed to the low levels of AFs. Drying maize storage in optimal conditions contributes to AF control [35]. If the low prevalence of aflatoxin-contaminated samples reflects the effective implementation of good practices by farmers, traders, and mills, this is an encouraging outcome for Nampula Province, which has previously been recognised as a hotspot for aflatoxin contamination [41]. However, the elevated contamination levels observed in some samples highlight the need for targeted monitoring and intervention strategies tailored to each stakeholder group within the supply chain and to the specific conditions of each district.

To control AF intake in food and prevent foodborne diseases, the Codex Alimentarius (Joint FAO/WHO Food Standards Programme) established a maximum level of 15 µg/kg for maize grain and 10 µg/kg for flour, meal, semolina, and flakes derived from maize [10]. The European Union set maximum levels of 5 µg/kg for AFB_1_ and 10 µg/kg for Total AF in maize that is to be subjected to sorting or other physical treatment before human consumption or use as an ingredient in foodstuffs [42]. Mozambique adopted a maximum level of 10 µg/kg for Total AF in food. In many African countries, regulations are ineffective [30]. To monitor AF levels in food throughout the value chain, a network of laboratories is needed to help farmers, traders, and mills in testing their products. Mozambique has limited access to laboratories, and test costs are high. The lack of laboratories and the high cost of testing in Africa create a bottleneck in monitoring AFs throughout the food supply chain [43].

Given that aflatoxin contamination of food and feed can pose significant risks to human and animal health and result in considerable economic losses, an integrated approach to aflatoxin management is essential throughout the maize supply chain. All stakeholders should adopt appropriate good agricultural and post-harvest practices to minimise aflatoxin contamination and ensure the production of safe, high-quality maize that meets health and market requirements.

## 4. Conclusions

This study found a low prevalence of aflatoxin (AF) contamination in maize samples collected from farmers, traders, and mills across all districts in 2024, while no AF was detected in 2025, indicating a promising outlook for Nampula Province. Annual temperature variation, AEZ, and Aflasafe use may have influenced aflatoxin levels. Among the contaminated farmer samples from 2024, a substantial proportion exceeded the Mozambican regulatory limit of 15 µg/kg, with levels in Mecuburi and Monapo reaching extreme concentrations. In addition, targeted Aflasafe deployment and farmer training in this district should be prioritised as an immediate intervention. Overall, coordinated action by farmers, traders, mills, public health authorities, and policymakers is necessary to reduce AF exposure, protect consumers, and safeguard the economic sustainability of the maize sector in Mozambique. To provide a broader assessment, similar studies should be extended to the remaining 18 districts of Nampula Province and to Mozambique more generally, incorporating all components of the maize value chain.

## 5. Materials and Methods

### 5.1. Study Area

Nampula Province, located in northern Mozambique, is the most populous, with more than 6 million inhabitants and has 23 districts. The Province is largely crossed by the R7 and R8 agro-ecological zones and slightly by the R10. Nampula districts selected for the study were Malema, Mossuril, Monapo, and Mecuburi for farmers, and Nampula County for traders and mills. Malema and Mecuburi are among the largest maize-producing districts in the inland region; Mossuril is among the largest maize-producing districts in the coastal region, followed by Monapo. Nampula is the capital city and major market in the northern region. The trials were conducted in two consecutive years, from May to June 2024 and 2025 during harvest time for farmers and from June to August 2024 and 2025 during marketing time for traders and mills.

### 5.2. Sampling

#### 5.2.1. Farmer Level

Maize samples were collected from fields managed by farmers cultivating at least 1 ha under monocropping systems. Sampling was conducted when the maize had reached physiological maturity and was ready for harvest. The study covered four districts of Nampula Province: Malema, Mossuril, Monapo, and Mecuburi.

Farmer sampling was based on updated lists provided by the District Economic Activities Services (SDAE) in each district. Farmers/households were selected using probability proportional to size (PPS) sampling to ensure that the sample allocation was representative of the farmer population in each district. Each year, 30 farmers were selected from Malema, 13 from Mossuril, 13 from Monapo, and 17 from Mecuburi. For laboratory analysis, samples were aggregated (Table 5). Nine maize cobs were randomly collected from different locations within each farmer’s field. The collected cobs were placed in polypropylene bags and transported under dry and cool conditions to the Laboratory of Lúrio University (CEIL-LQSA), Nampula, for further analysis. Before storage, maize cob samples were air-dried to a moisture content of 11% and 13%. The maize was then husked, threshed, and stored in 500 g sterile bags at 4 °C before being sent to the Central Analytical Facilities at Stellenbosch University (South Africa) for aflatoxin analysis.

During the sample collection, fields that applied Aflasafe, fertilisers, and pesticides were registered.

#### 5.2.2. Trader and Mill Level

Maize traders were randomly selected at Waresta Market, the largest wholesale market in Nampula City. Two years’ samples were collected from retailers (22) and wholesalers (14). The study also planned to collect 4 samples from exporters, but none were found. Mills’ samples were also collected in Nampula City. Four (4) samples were collected from medium-scale and large-scale mills each year. The samples from traders and mills were aggregated for laboratory analysis (Table 5). The large maize piles from traders and mills were systematically sampled by taking small quantities from multiple locations, including the bottom and top layers, from left to right, and along diagonal points. The subsamples were thoroughly combined to obtain a representative composite sample of approximately 1 kg for laboratory analysis. For small piles (less than 10 sacks), samples were taken from all bags in small quantities from the bottom, middle, and top of each bag to make a 1 kg aggregate sample. All samples (for traders and mills) were packed in sterile paper bags and transported to the laboratory under cool, dry conditions. Before storage, the samples were homogenised, divided into two parts, and stored at 4 °C before being sent to the Central Analytical Facilities at Stellenbosch University in South Africa for aflatoxin analysis.

### 5.3. Moisture Measurement

The moisture content of maize samples from farmers was recorded during two years (2024 and 2025) using a portable grain moisture tester (FARMEX MT-16, Farmcomp Oy, Tuusula, Finland) with high accuracy (±0.5%). Initial measurements were performed immediately after sample collection. Subsequently, the maize cob samples were air-dried (5 to 7 days) until their moisture content reached 11 and 13%, after which a second measurement was taken before storage.

### 5.4. Aflatoxin Analysis

#### 5.4.1. Chemicals and Standards

A 20 mg/L standard mixture of Aflatoxins B_1_, B_2_, G_1_ and G_2_ was purchased from CPI International (Santa Rosa, CA, USA). HPLC-grade methanol and acetonitrile solvents (Romil-SpSTM Super Purity) were purchased from Microsep (Sandton, South Africa). Milli-Q water was supplied by a Walters Mass-Directed Auto-Purification & QC System. Analytical Grade Formic Acid (Supelco, >98%) was purchased from Merck (Modderfontein, South Africa).

#### 5.4.2. Sample Grinding and Extraction

For each maize sample, a 200 g subsample was ground using a coffee grinder (Platinum, product of Popular Republic of China, special packed for Shoprite Checkers (Pty) Ltd., Brackenfell, Republic of South Africa). The blender and other materials were cleaned with 70% ethanol using lab tissue and allowed to air-dry before the next sample was processed to avoid cross-contamination among samples. After grinding, 1 g of each sample was weighed into a 50 mL tube, followed by the addition of 5 mL of extraction solvent (50% water: 25% methanol: 25% acetonitrile). The samples were vortexed thoroughly and sonicated in an ultrasonic bath (0.5 Hz, Integral Systems, RSA, Johannesburg, South Africa) for 1 h. After sonication, the samples were vortexed, and 2 mL of each sample was transferred into respective 2 mL Eppendorf tubes and centrifuged for 5 min at 13,000 rpm (Hermle Z160m, HERMLE Labortechnik GmbH, Wehingen, Germany). The supernatant layers were then double-diluted with 75%:25% water:methanol solvent and transferred into analysis vials.

#### 5.4.3. Calibration Standards and Quality Control Samples

Solvent calibration standards in the range of 0.3–1000 µg/L were made from the Aflatoxin B_1_, B_2_, G_1_, G_2_ standard mix (20 mg/L) using the extraction solvent, 25% methanol:25% acetonitrile:50% Milli-Q water. Quality control (QC) samples were prepared by spiking three respective solvent (50% water: 25% methanol: 25% acetonitrile) samples at three concentration levels: 7, 70 and 700 µg/L.

#### 5.4.4. Instrumental and Analytical Conditions

UPLC-MS/MS analysis of the aflatoxins was performed on a Waters Acquity Ultra-Performance Liquid Chromatograph (UPLC I—Class) connected to a Waters Xevo-TQS Micro Mass Spectrometer (Waters, Millford, MA, USA). Chromatographic separation was obtained using a Waters Acquity UPLC BEH C18 column (2.1 × 100 mm; 1.7 µm particle size) at 50 °C. An injection volume of 3 µL was used. The mobile phase consisted of 0.1% formic acid (solvent A) and 0.1% formic acid in acetonitrile (solvent B), and the following mobile phase gradient program was used: 90% A, 0–4 min; 33% A, 4–4.1 min; 0% A, 4.1–5.1 min; 90% A, 5.1–6.00 min.

Detection of analytes was performed using positive mode electrospray ionisation with multiple-reaction monitoring under the following conditions: capillary voltage, 3 kV; desolvation temperature, 450 °C; source temperature,150 °C; desolvation gas, 850 L/Hr; cone gas, 150 L/Hr. Table 6 displays the MS/MS settings used for multiple-reaction monitoring of the aflatoxins.

#### 5.4.5. Method Validation

The UPLC-MS/MS method was validated by evaluating the limits of detection (LOD) and quantification (LOQ), linearity, trueness (calculated as percent recovery) and precision (intra- and inter-day precision, calculated as the percent relative standard deviation, RSD). The solvent calibration standards were analysed in triplicate to establish the limit of detection (LOD), limit of quantification (LOQ) and linearity of the method. The LOD was determined as the lowest concentration of analyte that yielded a signal to noise (S/N) ratio of ≥3. The LOQ was determined as the lowest concentration that yielded a S/N ratio ≥ 10. The linearity of each analyte’s calibration standard was determined by the coefficient of determination (R2), which should yield a value of ≥0.98. The QC samples were analysed in quintuplicate. The accuracy (trueness) of the method was determined by calculating the average of the quintuplicate injections of the QC samples and measured as a percent recovery of the true value. The repeatability of the method was established by calculating the percent relative standard deviation (RSD) from the quintuplicate injections of the QC samples. Table 3 summarises the validation parameters evaluated for this method.

To assess the effect of the maize matrix on the detection and quantification of aflatoxins, QC samples were prepared in maize matrix at concentrations of 7, 70, and 700 µg/kg. The slope obtained from the plot of peak area versus concentration for the matrix-matched QCs were compared to the slope obtained from the calibration curve of the solvent standards to evaluate matrix effects. The effect of the maize matrix on the quantification of aflatoxins was concluded to be acceptable, as the percent matrix effect was less than 20% for all aflatoxins.

### 5.5. Statistical Analysis

Data were analysed using Microsoft Excel and R software (version 4.4.0). Descriptive statistics, including ranges, median, and IQR, were calculated for the quantitative variables. For AF measurements below the limit of detection (LOD), a value equivalent to half the LOD (LOD/2) was assigned for statistical analysis [44]. AF and moisture data for the two consecutive years were summarised and used in descriptive analysis to determine the mean values ± SD. AF values (µg/kg) were log-transformed *y* = log_10_ (1 + *x*) to normalise variances before being submitted to the Kruskal–Wallis test (95% confidence interval), with post hoc comparisons conducted using the Dunn test to assess differences in AF concentrations across districts. Statistical significance was set at *p* < 0.05. The Kruskal–Wallis test was used because the data did not meet the assumptions of normality and homogeneity required by the Analysis of Variance F-test.

## Figures and Tables

**Table 1:** Positive maize samples from farmers, traders and mills from two different years.

Year	Stakeholders	District	Number of Samples	Positive Samples	AFB_1_-Positive Samples (%)	AFB_2_-Positive Samples (%)	AFG_1_-Positive Samples (%)	AFG_2_-Positive Samples (%)	Samples > 5 µg/kg (%)	Samples > 15 µg/kg (%)
2024	Farmers	Malema	30 (10)	0	0	0	0	0	0	0
		Mossuril	13 (6)	2	17	0	17	0	34	17
		Mecuburi	17 (7)	3	43	43	29	29	43	43
		Monapo	13 (6)	1	17	17	0	0	17	17
	Traders	Nampula	18 (8)	1	13	13	0	0	13	13
	Mills	Nampula	8 (3)	0	0	0	0	0	0	0
2025	Farmers	Malema	30 (10)	0	0	0	0	0	0	0
		Mossuril	13 (6)	0	0	0	0	0	0	0
		Mecuburi	17 (7)	0	0	0	0	0	0	0
		Monapo	13 (6)	0	0	0	0	0	0	0
	Traders	Nampula	18 (8)	0	0	0	0	0	0	0
	Mills	Nampula	8 (3)	0	0	0	0	0	0	0

Note: Numbers in parentheses are aggregate samples submitted to aflatoxin analysis. >5 µg/kg = percentage of samples exceeding the Mozambique and EU maximum limit for AFB1 in food; >15 µg/kg = percentage of samples exceeding the Mozambican and Codex Alimentarius maximum total aflatoxin level in maize.

**Table 4:** Moisture content from farmers’ maize samples from the two years in the four Nampula districts.

Year	District	Number of Samples	Initial Moisture (%)		Final Moisture (%)	
			Mean ± SD	Range	Mean ± SD	Range
2024	Malema	30 (10)	13.94 ± 1.42	17.80–12.80	12.19 ± 0.62	13.40–11.20
	Mossuril	13 (6)	16.24 ± 0.77	17.10–15.30	12.06 ± 0.80	13.00–10.80
	Mecuburi	17 (7)	16.67 ± 1.20	17.70–14.70	12.63 ± 1.08	14.00–11.20
	Monapo	13 (6)	17.02 ± 0.74	17.80–15.80	12.60 ± 1.24	13.80–10.90
2025	Malema	30 (10)	14.71 ± 1.98	17.20–10.30	12.98 ± 1.34	15.40–10.30
	Mossuril	13 (6)	16.98 ± 0.79	17.50–15.80	12.00 ± 0.78	12.90–10.80
	Mecuburi	17 (7)	14.19 ± 1.08	15.80–12.90	12.51 ± 0.89	13.80–10.90
	Monapo	13 (6)	16.70 ± 1.10	17.70–14.70	12.60 ± 1.04	14.00–11.20

**Table 5:** Stakeholder sample numbers.

District	Stakeholder Number	Aggregated Samples	Missed Samples *
Malema	30	13	3
Mossuril	13	6	0
Mecuburi	17	7	0
Monapo	13	6	0
Nampula (traders)	18	8	0
Nampula (mills)	8	3	0
Total	99	43	3

* Missed samples are samples remaining in the inspection authority board during the quarantine.

**Table 6:** ESI-MS/MS parameters for the individual aflatoxins.

Name	Precursor Ion (*m*/*z*)	Product Ion (*m*/*z*)	Cone (V)	Collision (eV)
Aflatoxin B_1_	313	241	50	37
Aflatoxin B_1_	313	285	50	23
Aflatoxin B_2_	315	259	40	30
Aflatoxin B_2_	315	287	40	26
Aflatoxin G_1_	329	243	40	25
Aflatoxin G_1_	329	283	40	25
Aflatoxin G_2_	331	245.2	40	35
Aflatoxin G_2_	331	257	40	30

## Data Availability

The original contributions presented in this study are included in the article. Further inquiries can be directed to the corresponding author.
